# On the Use of Interaction Entropy and Related Methods to Estimate Binding Entropies

**DOI:** 10.1021/acs.jctc.1c00374

**Published:** 2021-07-13

**Authors:** Vilhelm Ekberg, Ulf Ryde

**Affiliations:** Department of Theoretical Chemistry, Chemical Centre, Lund University, P.O. Box 124, SE-221 00 Lund, Sweden

## Abstract

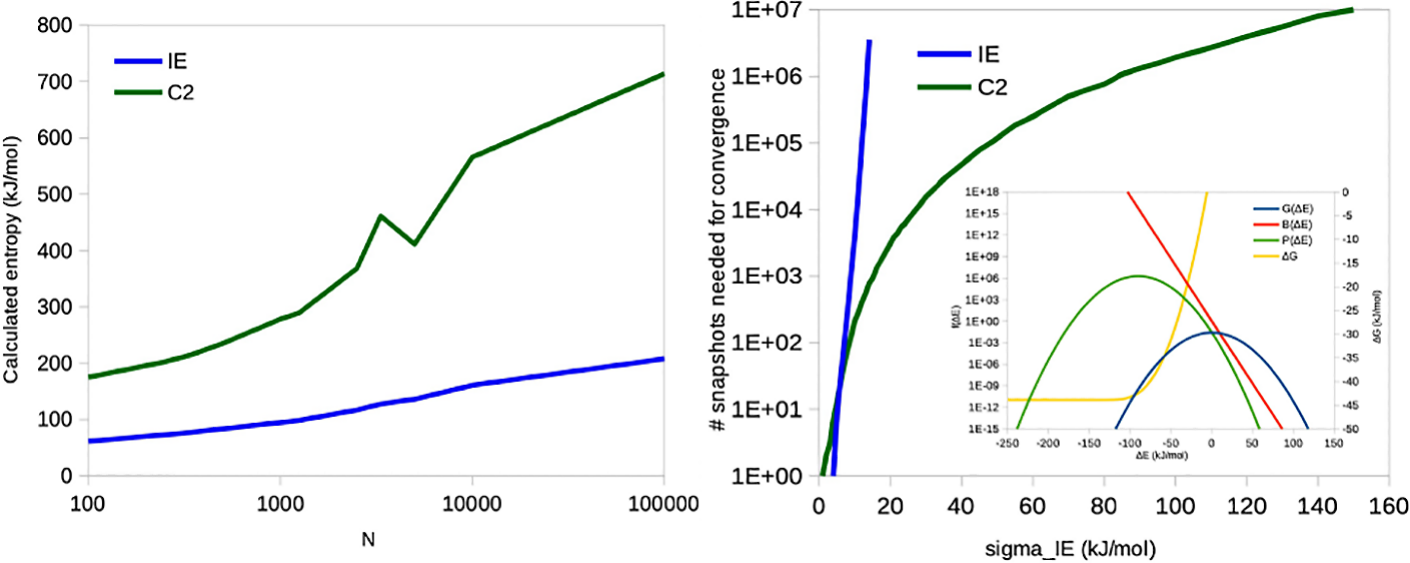

Molecular mechanics combined with Poisson–Boltzmann or generalized Born and solvent-accessible area solvation energies (MM/PBSA and MM/GBSA) are popular methods to estimate the free energy for the binding of small molecules to biomacromolecules. However, the estimation of the entropy has been problematic and time-consuming. Traditionally, normal-mode analysis has been used to estimate the entropy, but more recently, alternative approaches have been suggested. In particular, it has been suggested that exponential averaging of the electrostatic and Lennard–Jones interaction energies may provide much faster and more accurate entropies, the interaction entropy (IE) approach. In this study, we show that this exponential averaging is extremely poorly conditioned. Using stochastic simulations, assuming that the interaction energies follow a Gaussian distribution, we show that if the standard deviation of the interaction energies (σ_IE_) is larger than 15 kJ/mol, it becomes practically impossible to converge the interaction entropies (more than 10 million energies are needed, and the number increases exponentially). A cumulant approximation to the second order of the exponential average shows a better convergence, but for σ_IE_ > 25 kJ/mol, it gives entropies that are unrealistically large. Moreover, in practical applications, both methods show a steady increase in the entropy with the number of energies considered.

## Introduction

Estimating accurate free energies for the binding of small molecules to biomacromolecules is one of the most important goals of computational chemists because it would have a strong impact on drug development.^1−3^ Therefore, a large number of methods have been developed with this aim. Some methods involve docking using simplified scoring functions, which give fast, but not so accurate results.^4−6^ More accurate results are obtained with free-energy perturbation methods, which employ molecular dynamics (MD) simulations with standard force fields for the free and bound ligands, as well as for a number of alchemical intermediate states.^7−9^ Consequently, they are computationally very demanding, but they can give an accuracy of ∼4 kJ/mol for well-behaving cases.^10−12^

Intermediate between these two levels of theory, there are some methods that are also based on MD simulations but only of the physical end-states (the complex and possibly also the free protein and ligand).^13−15^ In particular, the molecular mechanics combined with Poisson–Boltzmann or generalized Born and solvent-accessible surface area (MM/PBSA and MM/GBSA) solvation energies have been much used.^16−18^ In these, the complex of the macromolecule and the ligand is simulated by MD simulations, and a number of snapshots are collected. For each of these, the water molecules are stripped off, and the binding free energy is approximated by
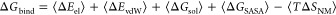
1where *E*_el_ is the electrostatic energy and *E*_vdW_ is the van der Waals energy, calculated with a standard MM force field, whereas *G*_sol_ is the solvation free energy calculated either by solving the Poisson–Boltzmann equation or by the Generalized Born approach, *G*_SASA_ is the non-polar solvation free energy, estimated from the solvent-accessible surface area (SASA), *T* is the absolute temperature, and *S*_NM_ is the translational, rotational, and vibrational entropy, estimated from a normal-mode (NM) analysis of vibrational frequencies calculated at the MM level of theory. Each energy term is estimated from the difference between the complex (RL), the free receptor (R), and the ligand (L)
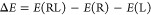
2normally obtained by simply stripping off the receptor or the ligand from snapshots taken from the MD simulations of the complex (otherwise the precision will be worse and there will be an additional energy term from the bond, angle, and dihedral interactions).^13−15^ Moreover, each energy term is an average over all snapshots from the MD simulation, indicated by the angular brackets in eq 1.

The time consumption of these approaches is often dominated by the Δ*S*_NM_ term (i.e., the frequency calculation), and Δ*S*_NM_ is therefore often calculated for only a fraction of the snapshots.^13−15,19,20^ However, then this term will limit the precision of the final results. As this term often does not improve the accuracy of the method (at least not the relative energies), it is often omitted. Several alternative approaches to estimate the binding entropy have been suggested.^19−22^

In 2016, Zhang and co-workers suggested a new method to estimate the binding entropy, called the interaction entropy (IE) approach.^23^ It estimates the entropy from
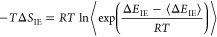
3where *R* is the gas constant and Δ*E*_IE_ = Δ*E*_el_ + Δ*E*_vdW_. Consequently, the entropies can be calculated directly from energies already available from the normal MM/GBSA (for simplicity, we will in the following say only MM/GBSA even when everything applies equally well for MM/PBSA) calculations and therefore does not add any extra computational cost. The IE method has been used in several later studies, also for protein–protein binding and alanine screening.^24−27^

In 2018, Minh and co-workers put the IE approach into a more general theoretical framework and tested a number of cheap methods to calculate the entropy.^22^ In particular, they expressed the binding free energy as an exponential average of Δ*E*_IE_ and approximated it by a cumulant expansion
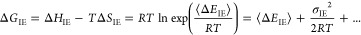
4where σ_IE_ is the standard deviation of *E*_IE_ over all snapshots. Consequently, the binding entropy can be approximated with the second-order cumulant approximation term (C2)
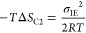
5In a recent study, we tried to use these approaches to estimate MM/GBSA binding free energies for the binding of three similar ligands to galectin-3^28^ but obtained poor and confusing results. Here, we explain those results and compare entropies obtained with the IE and C2 methods. In particular, we address the important question: how many snapshots are needed to obtain a converged estimate of the entropy with the IE and C2 methods? We answer the question by performing stochastic simulations, assuming that *E*_IE_ follows a Gaussian distribution, as has been done before for related questions.^29,30^

## Methods

### MD Simulations

We have studied the binding of five different ligands to three proteins: galectin-3 with three ligands, differing only in the position of a single fluorine group (*o*-, *m*-, and *p*-fluoro-phenyltriazolyl-galactosylthioglucoside, called O, M, and P in the following), ferritin with phenol, and the T4 lysozyme Leu99Ala mutant with benzene. All three systems have been studied before by us, and we used the same setup as in our previous studies (which therefore shows some slight variations).^28,31,32^ The simulations were based on the crystal structures of the complexes: 6RZF, 6RZG, 6RZH,^28^3F39,^33^ and 181L.^34^ All crystal-water molecules were kept in the simulations. Each complex was solvated in an octahedral (galectin-3 and lysozyme) or rectangular (ferritin) box of water molecules extending at least 10 Å from the protein using the *tleap* module. The protonation state of all residues is specified in our previous studies.^28,31,32^ No counter ions were used in the simulations.^35^

The MD simulations were run with the Amber software suite.^36^ The protein was described by the Amber ff14SB force field,^37^ water molecules with the TIP3P (ferritin and lysozyme) or TIP4P-Ewald model (galectin-3),^38^ whereas the ligands were treated with the general Amber force field (GAFF).^39^ Charges for the ligands were obtained with the restrained electrostatic potential method,^40^ and they were specified before.^28,31,32^

For each complex, 1000 steps of minimization were used, followed by 20 ps constant-volume equilibration and 20 ps constant-pressure equilibration, all performed with heavy non-water atoms restrained toward the starting structure with a force constant of 4184 kJ/mol/Å^2^. The system was then equilibrated freely for 1 ns. Two sets of production simulations were then performed for each of the five complexes with constant pressure and without any restraints: In the first, we run 100 ns simulation and sampled snapshots every 10 ps. In the second, we run 10 ns simulation and sampled snapshots every 10 fs. The former is similar to what we normally do in ligand-binding studies,^28,31,32,41^ whereas the latter is more similar to what was done in the original IE publication.^23^ In both cases, we run 10 independent simulations for each system, using different starting velocities and water solvation boxes.^42^ Consequently, the total simulation time for each complex was 1 μs and 100 ns, respectively, and we collected *N* = 100,000 and 10,000,000 snapshots from each simulation.

All bonds involving hydrogen atoms were constrained to the equilibrium value using the SHAKE algorithm,^43^ allowing for a time step of 2 fs. The temperature was kept constant at 301 K (galectin-3 and lysozyme) or 298 K (ferritin) using Langevin dynamics,^44^ with a collision frequency of 2 ps^–1^. The pressure was kept constant at 1 atm using a weak-coupling isotropic algorithm^45^ with a relaxation time of 1 ps. Long-range electrostatics were handled by particle-mesh Ewald summation^46^ with a fourth-order B spline interpolation and a tolerance of 10^–5^. The cutoff radius for Lennard-Jones interactions was 8 Å (galectin-3 and lysozyme) or 10 Å (ferritin).

### MM/GBSA Calculations

MM/GBSA calculations^16−18^ were performed using mmpbsa.py utility of AMBER.^47^ The calculations employed the latest generalized Born method GB-Neck2 (igb = 8) with modified Bondi radii (mbondi3)^48^ and a dielectric constant of 80 outside the solute and 1 inside the solute. The non-polar solvation free energy was calculated from the solvent accessible surface, using Δ*G*_SASA_ = α SASA + *b*, with α = 0.0227 kJ/mol/Å^2^ and *b* = 3.85 kJ/mol.^49^

Entropies were calculated by the IE^23^ and C2 approaches^22^ using the simulated data and a local script. We also divided the complete data (all snapshots) into smaller batches, allowing for estimates of the precision of the estimated entropies (as the standard deviation over the calculated entropy for each batch divided by the square root of the number of batches of equal size).

In addition, we also discuss results of previous MM/GBSA calculations on avidin with seven biotin-like ligands,^41^ blood clotting factor Xa with nine inhibitors,^50^ galectin-3 with two additional ligands,^51^ and ferritin with eight additional small ligands.^31^ Using the old data, we have calculated IE and C2 entropies with the same script. Most of the old studies reported also entropies estimated with the NM approach.

All entropies in this article are discussed in energy terms, that is, as −*T*Δ*S* in kJ/mol at 300 K.

### Gaussian Simulations

The stochastic simulations used the same approach as in our previous study of the convergence of exponential averaging to estimate reaction free energies in combined quantum and molecular mechanical calculations.^29^ In fact, the same small simulation program could be used, besides that entropies were considered, instead of free energies (so that average of Δ*E*_IE_ was not included). The program generates a certain number of Gaussian-distributed energies (by the Box–Muller transform^52^) and calculates the exponential average in eq 3. This is repeated 1000 times, and it is noted how many times this average is within a certain limit (we used 4 kJ/mol for all calculations in this study) from the analytical results (eq 5; which is the analytical result for a Gaussian distribution). The program automatically finds the minimum number of snapshots (*N*, estimated within 0.1%) needed to fulfil these criteria. The Fortran code can be obtained from the authors upon request.

## Results and Discussion

### Galectin-3

In a recent investigation of the binding thermodynamics of three ligands to galectin-3,^28^ we were suggested by a reviewer to estimate the energy and entropy of binding using the MM/GBSA and IE approaches. This was an attractive suggestion because we already had 10 × 100 ns simulations of the protein–ligand complexes with structures sampled every 10 ps, so it was only a matter of postprocessing of these snapshots. Unfortunately, the results were not especially encouraging and were therefore presented only in the Supporting Information.^28^

**Figure 1:**
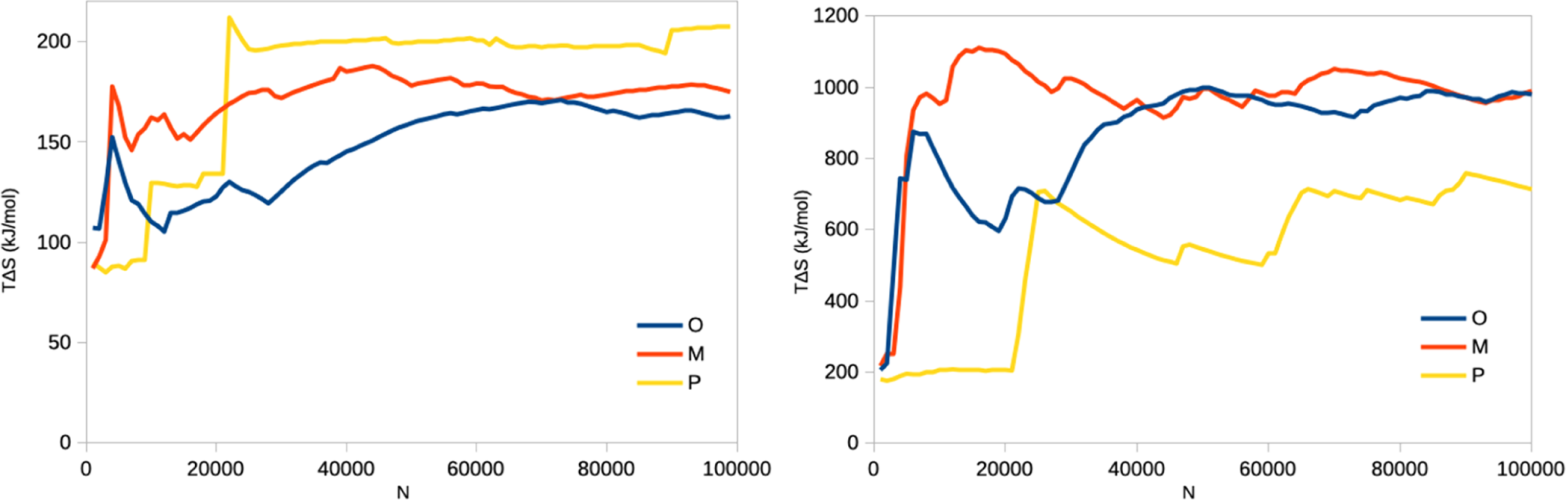
Running average of IE (left) and C2 (right) entropies for the binding of three ligands (O, M, and P) to galectin-3 as a function of the number of snapshots from the 10 × 100 ns simulations with a sampling frequency of 10 ps.

In particular, whereas the MM/GBSA energies were reasonably well-converged, the interaction entropies showed a very alarming trend depending on how the entropies were calculated: If all data were used for exponential average in eq 3 (*N* = 100,000, the number of snapshots and individual Δ*E*_IE_ energy estimates), we obtained very large entropies for all three ligands, 162–208 kJ/mol, giving positive binding free energies of 33–56 kJ/mol (the experimental estimates are −30 to −33 kJ/mol^28^). The results look reasonably converged with a variation of 2–5 kJ/mol for the running average over the last 10% of the snapshots, as can be seen in Figure 1.

These entropies do not have any estimates of the uncertainty, and it is more natural to calculate interaction entropies for each of the 10 independent simulations separately (*N* = 10,000 in each simulation), presenting the average entropy and the standard error over the 10 sets of simulations. This gave somewhat lower estimates, 160 ± 9, 161 ± 9, and 153 ± 10 kJ/mol, for O, M, and P, respectively, but still positive binding free energies (9–25 kJ/mol).

**Figure 2:**
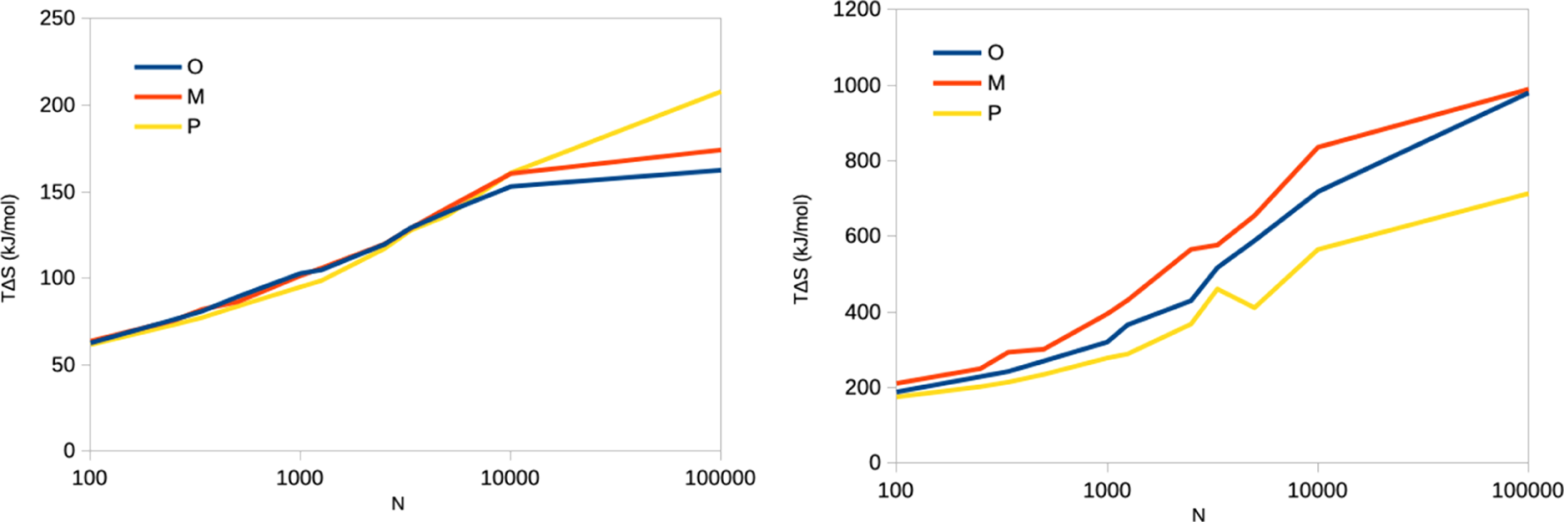
Entropies estimated by the IE (left) and C2 (right) methods by block averaging for the binding of three ligands to galectin-3 as a function of *N* from the 10 × 100 ns simulations. Note the logarithmic scale on the *x*-axis.

However, we have previously found that more stable entropies are obtained (by dihedral histogramming) if they are obtained from simulations of 5 ns (*N* = 500; the entropies are averaged over 200 batches) because it reduces the dependence on rare events.^53^ The 5 ns time window was selected to be similar to the rotational correlation time of the protein (∼7 ns).^28^ Quite surprisingly, this gave much smaller entropies, 84–89 kJ/mol, with a standard error of 2 kJ/mol. We therefore repeated the calculations with *N* varying from 100 to 100,000. From Figure 2a, it can be seen that the estimated IE entropy increases steadily with *N*. At the same time, the estimated standard error increases from 0.5 to 0.6 kJ/mol at *N* = 100 to 9–10 kJ/mol at *N* = 10,000, reflecting that the entropies are averaged over fewer independent simulations (from 1000 to 10; the range of the estimated entropies is actually larger at *N* = 100 than at *N* = 10,000, 140–210 kJ/mol, compared to 83–97 kJ/mol).

Naturally, these results are very alarming, showing that we can essentially get any estimate of the entropy between 60 and 200 kJ/mol. To yield some further understanding, we calculated entropies also with the second-order cumulant approximation, C2, as suggested by Minh and co-workers.^22^ The results are shown in Figures 1b and 2b, and it can be seen that it gives much higher entropies, 175–990 kJ/mol, but with the same increasing trend with respect to *N*.

**Figure 3:**
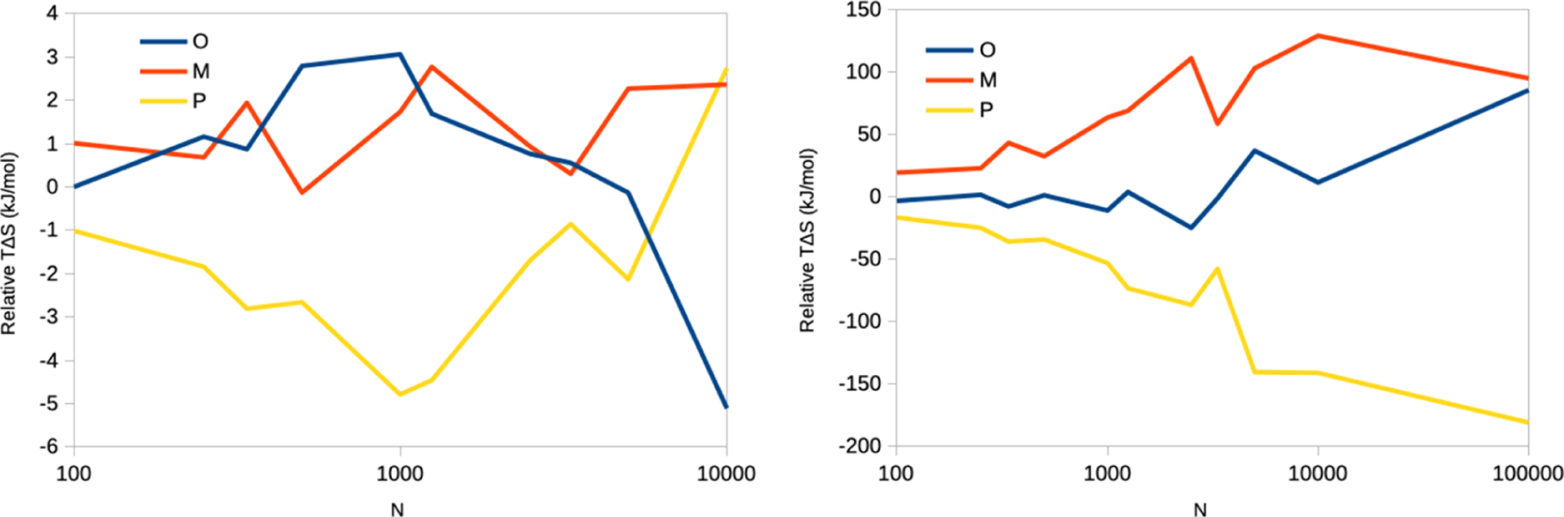
Relative entropies (defined as the entropy of each ligand minus the average of the three ligands) estimated by the IE (left) and C2 (right) methods for the binding of three ligands to galectin-3 from the 10 × 100 ns simulations. For IE, points with *N* = 100,000 (−19, −7 and 26 kJ/mol for O, M, and P, respectively) were omitted to emphasize the variation for the other, more precise estimates.

It could be hoped that *relative* entropies are more stable. Therefore, we show in Figure 3 the relative entropy of the three ligands as a function of *N*. It can be seen that the C2 entropies show a consistent trend (P < O < M) for all values of *N*, although the range (the difference between the largest and the smallest value) increases from 36 to 280 kJ/mol. However, IE entropies do not give any consistent results, although P has the smallest entropy for *N* ≤ 5000. The range of the entropies increases from 2 to 45 kJ/mol.

We also repeated the entropy calculations from 10 × 10 ns simulations with a sampling frequency of 10 fs (i.e., more similar to what was used in the original IE study^23^). However, they gave similar results, as can be seen in Figures S1 and S2. Moreover, we tried to remove various amounts of the initial part of the simulation as equilibration (in Figure 2, the equilibration time was 1 ns). However, this did not lead to any qualitative change in the results (Figure S3 shows the results when 51 ns of equilibration was used).

Next, we studied the root-mean-squared deviation (rmsd) of the ligand from the starting (crystallographic) conformation. It can be seen from Figure S4 that it in general fluctuates around 1.2 Å. However, occasionally, it increases to higher values (up to 5.5 Å). For a few simulations, it also stabilizes around 3 Å. These fluctuations do not represent full unbinding of the ligand but a change in the conformation of parts of the ligand. Still, it is likely that these conformational changes may affect the estimated entropies. Minh and co-workers suggested that only conformations with a low rmsd should be used for the entropy calculations,^22^ and we therefore tested to exclude all snapshots with a ligand rmsd > 2.2 Å. The results in Figure S5 show that it did not improve the convergence of the entropies.

Finally, we have also tried to extrapolate the block-averaged IE entropies with a power series in 1/*N*, following the suggestion by Zuckerman and co-workers.^54,55^ However, as can be seen in Table S1, the extrapolations give a large uncertainty and a strong dependence on the exponent of the power series.

### Curse of Exponential Averaging

At first, we assumed that we made some error in the calculations, owing to the large discrepancy between the IE and C2 results, but after some further considerations, we convinced ourselves that the calculations are correct and the large discrepancy actually explains the problem. In fact, we had already given the explanation before but in another context.^30^ Several other groups have also pointed out the poor convergence of exponential averaging.^54−58^

If Δ*E*_IE_ follows a Gaussian distribution, the last sum in eq 4 truncates at the second term, and the IE and C2 estimates should coincide. Owing to the central limit theorem, it is reasonable to assume that most Δ*E*_IE_ data should be approximately Gaussian (ref (22) shows distributions for 87 protein–ligand complexes, supporting this suggestion). Therefore, a powerful technique to judge the performance, convergence, accuracy, and precision of methods like IE is to assume that Δ*E*_IE_ follows a Gaussian distribution and perform numerical simulations with random Gaussian-distributed data.^29^ With such a simulation, it is simple to show that as long as the standard deviation of the Δ*E*_IE_ energies, σ_IE_, is small, Δ*S*_IE_ and Δ*S*_C2_ indeed coincide.

Moreover, we can answer the question: how many independent Δ*E*_IE_ energies (*N*) are needed to obtain a reliable estimate of the entropy, which for a Gaussian distribution is the C2 estimate. We only need to specify what we mean by “reliable.” Following our previous study,^30^ we define reliable as giving an entropy within 4 kJ/mol of the analytical results with 95% confidence (but other values could easily be used). 4 kJ/mol corresponds to a factor of 5 in the binding constant, which seems to be a proper limit for a reliable estimate.

**Figure 4:**
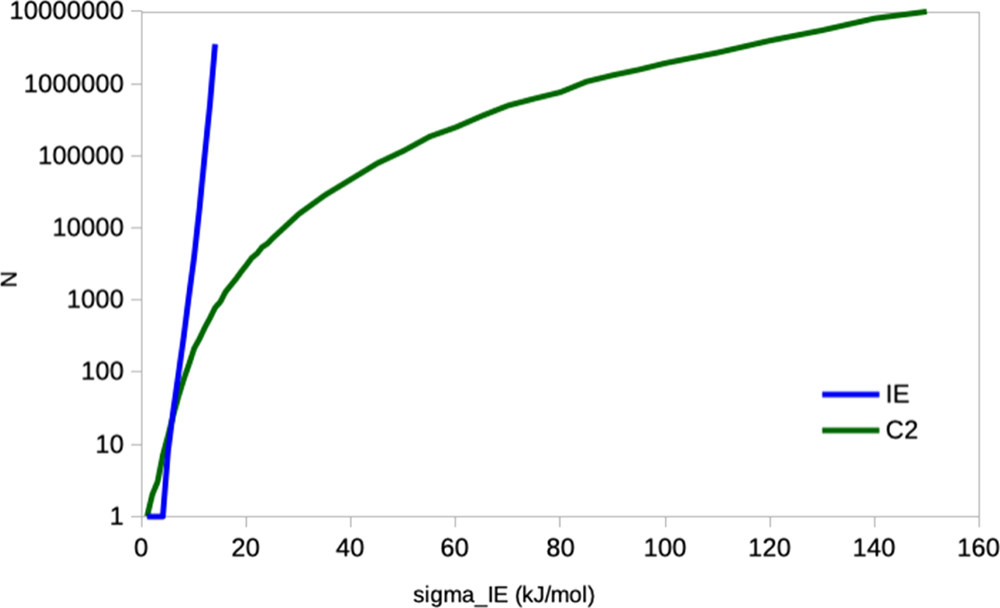
Number of snapshots (*N*) needed to converge the IE and C2 entropies within 4 kJ/mol of the analytical result with 95% confidence, assuming that the Δ*E*_IE_ energies follow a Gaussian distribution.

The results of the simulations are shown in Figure 4. It can be seen that for σ_IE_ < 10 kJ/mol, the IE entropy converges smoothly and only a rather small number of snapshots is needed (e.g., *N* = 1100 for σ_IE_ = 9 kJ/mol). However, when σ_IE_ > 10 kJ/mol, the convergence rapidly deteriorates and *N* increases exponentially. In practical applications, the upper limit is around *N* = 10,000,000, at which point the size of the coordinate files becomes several TB, even after stripping of the water molecules. This limit is reached around σ_IE_ = 15 kJ/mol. If σ_IE_ is larger than that, it will be practically impossible to converge the IE entropy, and the IE estimates will gravely underestimate the true entropy and will increase as the sampling is increased, as observed in Figure 2 (for which σ_IE_ = 60–70 kJ/mol).

**Figure 5:**
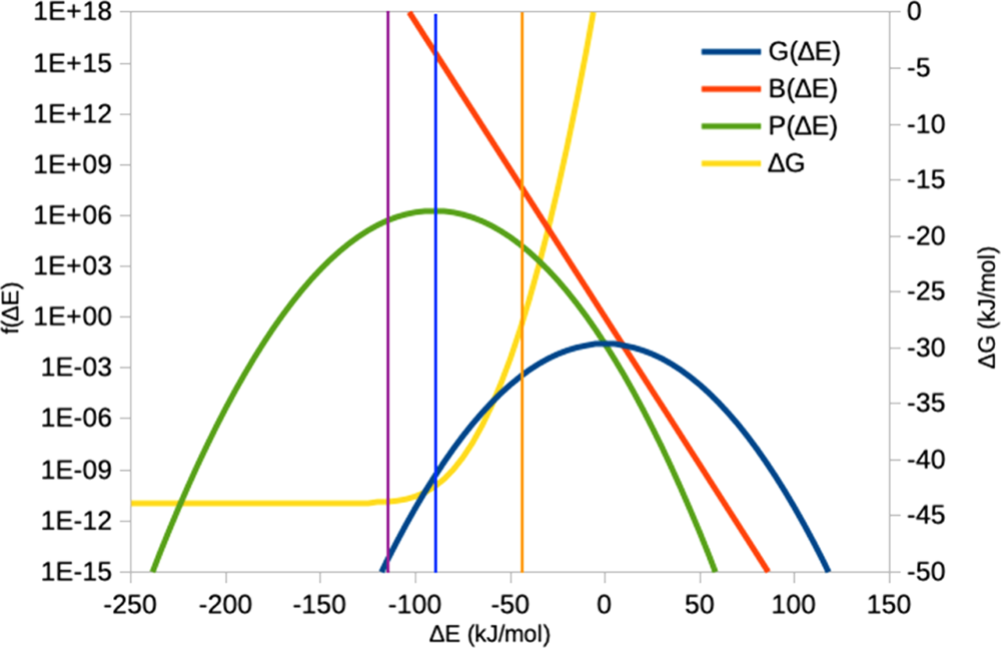
Problem of converging the exponential average. Assuming that Δ*E* follows a Gaussian distribution, the exponential average can be rewritten as an integral over the product of two terms , the Gaussian distribution  and the Boltzmann factor .^30^ These two terms are shown in the figure for the example of σ_IE_ = 15 kJ/mol (note the logarithmic scale). In this case, the maximum for the product is attained at Δ*E* = 90 kJ/mol (blue vertical line). At this value, *G* = 4 × 10^–10^ (but *B* = 5 × 10^15^), so around 10^10^ snapshots are needed before this value is observed. In fact, Δ*G* and therefore Δ*S* (which is Δ*G* minus the average of Δ*E*) are still not fully converged (Δ*G* in the figure; right axis; it should be read from the right to the left, i.e., showing Δ*G* when all values larger than Δ*E* are included), differing by 1.4 kJ/mol from the analytic result; convergence to within 0.1 kJ/mol is obtained at Δ*E* = −115 kJ/mol, when *G* = 5 × 10^–15^ (violet vertical line). If all Δ*E* < 3σ_IE_ (= −45 kJ/mol) are ignored, Δ*G* and *T*Δ*S* will be wrong by over 15 kJ/mol (orange vertical line) because the most important parts of *P* are excluded.

The reason for this is that the exponential average is extremely badly conditioned.^30^ The exponential average depends critically on energies that give the largest value of the exponential in eq 3. If the standard deviation of the distribution, σ, is small, these values are rather likely and are therefore frequently found in a simulation. However, as σ increases, the most important values become extremely rare and may actually never be observed in a MD simulation of a normal length. Consequently, the exponential average will typically underestimate the true result (obtained by an infinite number of snapshots, or by assuming that the distribution is indeed Gaussian, in which case the exponential average should converge to the second-order cumulant result). This is illustrated in Figure 5.

Fortunately, the C2 estimate has a better convergence. For example, at σ_IE_ = 15 kJ/mol, less than 1000 energies are needed. At σ_IE_ = 25 kJ/mol, still only 7200 snapshots are needed, but on the other hand, the estimated entropy starts to be very large, −*T*Δ*S*_C2_ = 125 kJ/mol, indicating that other approximations start to break down. The results in Table 1 (to be discussed below) show that NM entropies typically are 52–126 kJ/mol, and Duan et al. report NM entropies of 0–122 kJ/mol.^23^

**Table 1:** Values of σ_IE_, *T*Δ*S*_IE_, *T*Δ*S*_IE_, *T*Δ*S*_IE_, and IE from Our Previous MM/GBSA Investigations^28,31,41,42,50,51^ and in the Present Study^a^

protein	ligand	length	*f*	σ_IE_	*–T*Δ*S*_IE_	*–T*Δ*S*_C2_	*–T*Δ*S*_NM_
lysozyme^42^	Bz	20 × 0.2	5	6	6	7	52
		40 × 0.2		6	18	6	53
b		10 × 10	0.01	6	15	6	
b		10 × 100	10	6	14	7	
ferritin^31^	L1	40 × 1.2	10	9	15	18	67
	L2			11	25	26	73
	L3			10	18	20	70
	L4			10	19	20	68
	L5			12	21	28	63
	L6			10	16	20	60
	L7			10	14	19	59
	L8			10	13	19	56
	L9			13	19	34	82
b	L1	10 × 7.5	0.01	13	23	33	
b		10 × 100	10	13	24	34	
galectin-3^28^^,^^b^	O	10 × 10	0.01	54	185	583	
	M			46	199	428	
	P			40	206	328	
	O	10 × 100	10	70	162	980	
	M			70	174	990	
	P			60	208	714	
galectin-3^51^	Lac	10 × 200	100	37	161	274	126
	L02			30	152	177	122
	Lac	40 × 0.2	5	45	190	398	99
	L02			49	185	476	100
fXa^50^	CBB	40 × 0.2	5	66	154	880	114
	C9			61	159	737	116
	C39			40	119	321	116
	C47			66	167	872	118
	C49			69	208	949	119
	C50			71	254	1009	119
	C53			66	218	876	119
	C57			41	113	333	115
	C63			41	125	330	113
avidin^41^	Btn1	4 × 25 × 0.2	5	48	166	469	102
	Btn2	4 × 30 × 0.2		47	162	452	103
	Btn3	4 × 20 × 0.2		50	213	495	100
	Btn4	4 × 50 × 0.2		25	82	126	99
	Btn5	4 × 40 × 0.2		25	48	123	78
	Btn6	4 × 20 × 0.2		20	43	80	75
	Btn7	4 × 20 × 0.2		21	53	91	66
	Btn1	25 × 0.2		13	30	35	102
				13	38	36	101
				14	46	40	101
				13	42	32	102
	Btn2	30 × 0.2		14	40	41	103
				14	32	40	104
				15	40	44	102
				14	42	41	103
	Btn3	20 × 0.2		14	38	38	98
				14	38	40	100
				15	39	43	101
				15	56	48	101
	Btn4	50 × 0.2		13	29	33	100
				13	33	33	97
				13	38	36	99
				12	30	31	98
	Btn5	40 × 0.2		10	32	21	77
				13	33	33	78
				13	38	36	78
				12	30	31	79
	Btn6	20 × 0.2		10	18	19	74
				9	23	16	75
				10	18	18	75
				10	18	18	75
	Btn7	20 × 0.2		8	19	14	70
				8	15	14	67
				8	17	13	64
				9	22	16	68

a The table shows the protein, the ligand (named after the original publications), the length of the simulation (number of independent simulations times the length of the production simulation in ns; for avidin, an initial “4×” signifies that the four binding sites in the homotetrameric protein are considered at the same time) and the sampling frequency (*f* in fs).

b Present investigation.

It might seem strange to simulate the C2 entropy with a Gaussian distribution, for which the C2 estimate is exact. However, since the C2 entropy is estimated from the *square* of the standard deviation from the finite sample of Δ*E*_IE_ energies (eq 5), the random variation of the sampled values become quite large when σ_IE_ is large and therefore many snapshots are needed before the precision is good enough to reproduce the correct value of the C2 entropy within 4 kJ/mol with 95% confidence.

The conclusion from this exercise is that if σ_IE_ < 10 kJ/mol, both IE and C2 can be used to estimate the entropy, they should give identical results (within 4 kJ/mol) and ∼1000 snapshots can be used, as is typical for MM/GBSA. If 15 ≤ σ_IE_ < 25 kJ/mol, IE becomes impossible to converge; IE and C2 entropies will start to diverge and C2 is strongly to be preferred. For C2, it is still enough with a few thousands of snapshots. Finally, if σ_IE_ > 25, C2 can still be converged (up to σ_IE_ ≈ 150 kJ/mol), but the estimated entropy is most likely grossly overestimated.

### Comparison with Previous Results

To evaluate whether the results of our Gaussian simulations are representative, we examine the results of some of our previous MM/GBSA studies. The data are presented in Table 1 and are typically based on *N* = 1600–5000 snapshots. It can be seen that the magnitude of σ_IE_ depends mainly on the protein. Proteins with a buried binding site give a low σ_IE_, for example, lysozyme (6 kJ/mol), ferritin (9–13 kJ/mol), and avidin (8–15 kJ/mol), whereas proteins for which the ligand binds on the surface give larger σ_IE_, for example, factor Xa (40–71 kJ/mol) and galectin-3 (30–70 kJ/mol). However, there are also clear trends among the ligands. For example, for avidin, small and neutral ligands, like Btn6 and Btn7, give lower values of σ_IE_ (8–10 k/mol) than the larger and negatively charged ligands Btn1–Btn3 (13–15 kJ/mol). Moreover, the results also depend on the details of the simulation. For example, if all four binding sites of the tetrameric avidin protein are considered at the same time, σ_IE_ becomes much larger than if the sites in each subunit are considered individually.

**Figure 6:**
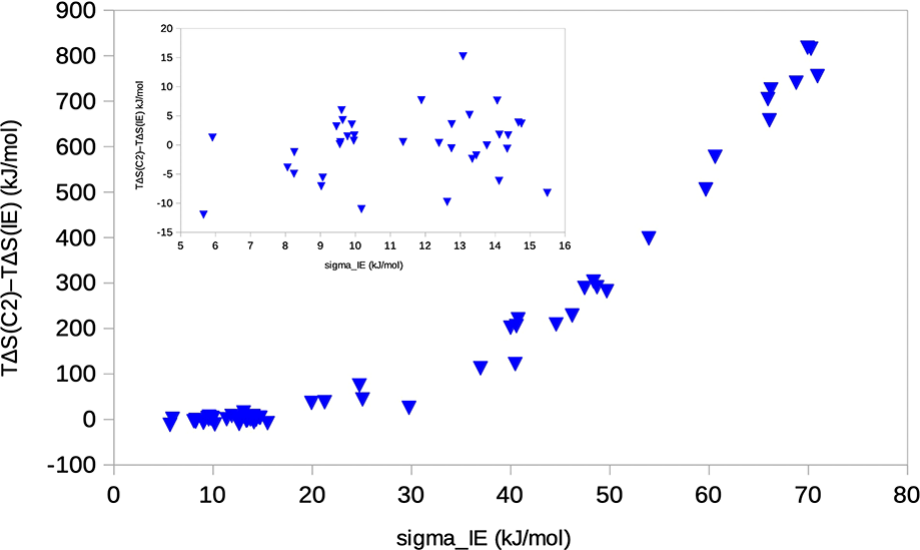
Difference between the C2 and IE entropies as a function of σ_IE_ for our previous MM/GBSA studies (raw data in Table 1). The inset shows the results with σ_IE_ < 16 kJ/mol.

It is hard to decide the convergence of the calculated entropies without doing additional simulations. However, a first indication can be obtained by comparing the IE and C2 entropies. The difference between these two estimates is shown as a function of σ_IE_ in Figure 6. It can be seen that for σ_IE_ < 16 kJ/mol, the IE and C2 entropies agree reasonably (within 15 kJ/mol) and with no consistent trend (inset in Figure 6). However, for larger σ_IE_ values, the difference between the two methods increases (essentially linearly for σ_IE_ > 35 kJ/mol), and the IE entropy is always smaller than the C2 entropy. This confirms our results in the previous section that it is completely impossible to obtain any reliable estimate of the IE entropy if σ_IE_ > 15 kJ/mol and that the C2 entropies are more accurate (but too large in magnitude).

Our previous MM/GBSA studies involved also estimation of the entropies with the NM method. There is a fair correlation between entropies calculated between the three methods: *R* = 0.62 and 0.72 between the NM and C2 or IE entropies, respectively. In fact, the correlation is very good (*R* = 0.86–0.89 for all three combinations) for lysozyme, ferritin, and avidin, and the NM entropies are always larger in magnitude by 35–72 kJ/mol (54 kJ/mol on average for both IE and C2). On the other hand, there is no correlation between NM and the other two methods (*R* < 0.12) for galectin-3 and factor Xa (but the IE and C2 entropies have a fair correlation of *R* = 0.68). In these cases, C2 entropies are the largest, IE entropies are intermediate, and NM entropies are the smallest (54 and 470 kJ/mol smaller than IE and C2 on average), but the variation is large.

Next, we consider the results in the study by Menzer et al., which implicitly reports σ_IE_ in their Figure S2.^22^ Among their 87 examined protein–ligand complexes, σ_IE_ varies between 6 and 42 kJ/mol (with an average of 17 kJ/mol). As in our study, the lowest values are found for lysozyme (6–8 kJ/mol). This shows that our estimates of σ_IE_ (6–71 kJ/mol) are comparable with σ_IE_ estimates in other studies and are not unusually large.

Likewise, we have examined the results in the original IE article.^23^ They examined 15 protein–ligand complexes with two simulation protocols. They do not report σ_IE_, but we can get an approximation of it from eq 5, but using *S*_IE_ instead of *S*_C2_ (it will underestimate σ_IE_ when it is larger ∼15 kJ/mol). This gives values for σ_IE_ of 8–24 kJ/mol, 43% of which are larger than 15 kJ/mol. In the first simulation protocol, the protein structure was restrained by a force constant of 42 kJ/mol/mol/Å^2^, and the simulation was run for 2 ns. In the second protocol, no restraints were used, and the simulation length was 6 ns. In both cases, Δ*E*_IE_ energies were sampled every 10 fs from the last 1 ns of the simulation (i.e., *N* = 100,000). The restrained simulations always gave a lower entropy by 1–11 kJ/mol, showing that the restraints are not innocent. On the other hand, it reduced the number of simulations with σ_IE_ > 15 kJ/mol from 67 to 20%. Minh and co-workers performed a systematic study of the effect of restraints on the simulations for 54 protein–ligand complexes for five proteins.^59^ They showed that the calculated entropies vary by up to 140 kJ/mol when the restraint weight varied. The entropies are 26 and 38 kJ/mol on average for IE and C2, with a difference of up to 119 kJ/mol (the average and maximum values of σ_IE_ are 14 and 34 kJ/mol). Naturally, *T*ΔS_IE_ is even larger for protein–protein interactions, for example, 171–287 kJ/mol, for 13 complexes studied by Zhang and co-workers.^24^

### Simulations of Lysozyme and Ferritin

To get some additional perspective of the performance of the IE and C2 methods, we run new simulations for two protein–ligand systems, benzene bound to the Leu99Ala T4 lysozyme mutant and phenol bound to a ferritin dimer. The systems were selected to illustrate cases where the two entropy methods are expected to work well (lysozyme with σ_IE_ = 6 kJ/mol) and where the performance could start to be problematic (ferritin with σ_IE_ ≈ 13 kJ/mol). In both cases, we run two sets of simulations (the same as for galectin-3), 10 × 10 ns simulations with a sampling frequency of 10 fs (*N* = 10,000,000) and 10 × 100 ns with a sampling frequency of 10 ps (*N* = 100,000). The pooled simulations were then divided into blocks of decreasing *N*, as for galectin-3.

**Figure 7:**
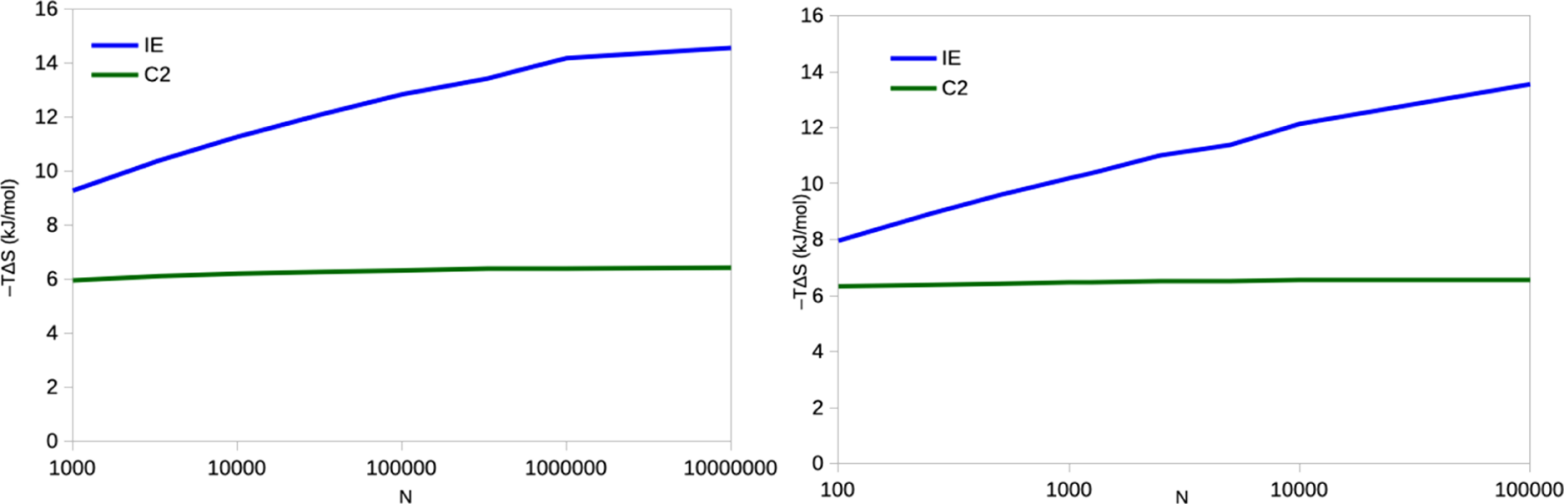
Entropies estimated by the IE and C2 methods for the binding of benzene to T4 lysozyme, estimated from 10 × 10 ns simulations with a sampling frequency of 10 fs (left) or 10 × 100 ns simulations with a sampling of 10 ps (right). In both cases, the simulations are then divided into batches of different sizes. Note the logarithmic scale on the *x*-axis.

The results for lysozyme are shown in Figure 7. For both sets of simulations, it can be seen that the C2 entropies are stable, with a variation of only 0.3–0.4 kJ/mol for the different batch sizes (reflecting a variation of the estimated σ_IE_ of 0.1–0.2 kJ/mol). On the other hand, the IE entropy increases by 5–6 kJ/mol as *N* is increased. Moreover, the estimated entropy is 0.7–0.9 kJ/mol lower when estimated from the simulations with the high sampling frequency (comparing results obtained with the same *N*). This probably reflects that the 10 fs sampling is too dense: the correlation of the Δ*E*_IE_ energies between two snapshots 10 fs apart is 0.74, whereas it is 0.04–0.06 between the snapshots 10 ps apart (0.31 for 0.1 ps and 0.07 for 1 ps). The difference goes down to 0.3 kJ/mol for a sampling frequency of 0.1 ps and to essentially zero for a sampling frequency of 1 ps. The estimated statistical efficiency is ∼3. In a previous study, we estimated the correlation time between different MM/GBSA energies to 1–10 ps.^41^ Sampling correlated energies will not affect the estimated entropies, only slow the convergence (in terms of *N* but not in terms of the total simulation time). For example, if we use each energy 10 times in the Gaussian simulation, 10 times more energies are needed to reach the same level of convergence for each value of σ_IE_, both for IE and C2 entropies.

**Figure 8:**
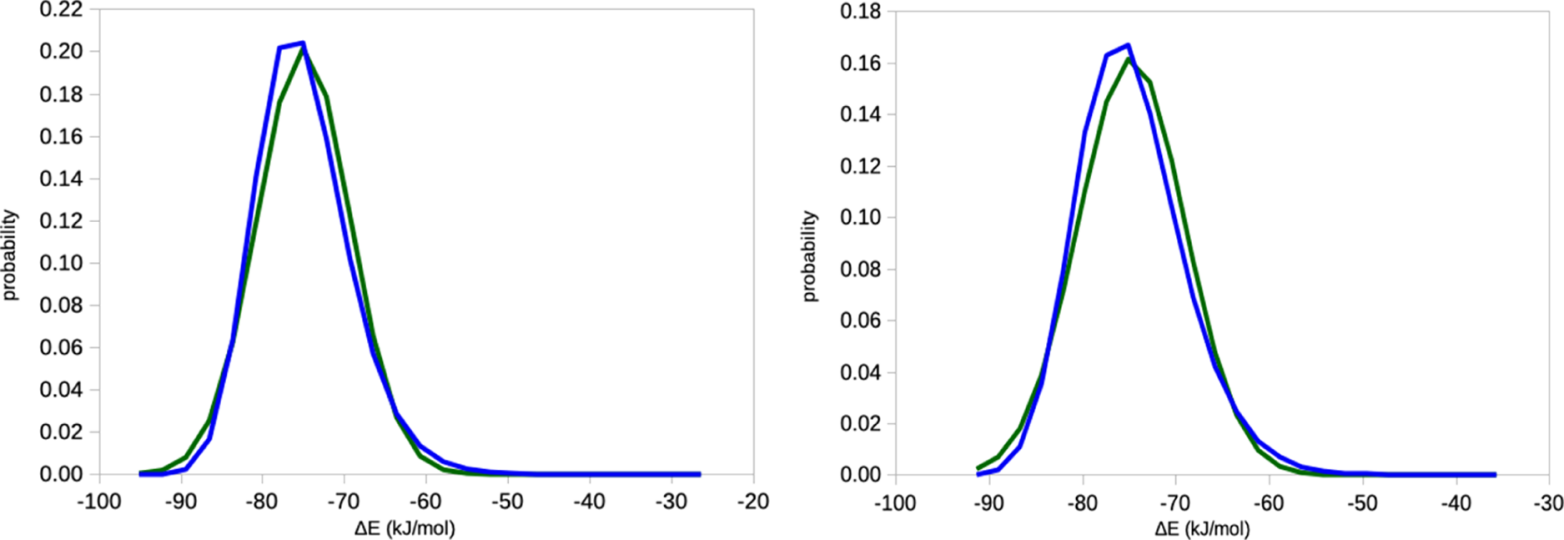
Distribution of the Δ*E*_IE_ energies for the binding of benzene to T4 lysozyme, estimated from 10 × 10 ns simulations with a sampling frequency of 10 fs (left) 10 × 100 ns simulations with a sampling of 10 ps (right). The green curve shows the ideal Gaussian distribution with the same average and standard deviation.

It should be noted that this rather strong dependence of the IE entropy on *N* is unexpected. If we instead base the calculations on Gaussian distributed random energies with the same mean and σ_IE_ as the simulated data, the calculated IE entropies vary by only 0.1 and 0.6 kJ/mol for the short and long simulations, respectively (and the C2 entropies by less than 0.01 kJ/mol). Thus, the variation seems to come from the fact that the Δ*E*_IE_ energies do not exactly follow a Gaussian distribution. This is confirmed by the distributions, as shown in Figure 8. It would then be tempting to prefer the IE results, but the steady increase in the IE entropy with *N*, without any sign of convergence, makes it problematic to use in practice.

We have examined the movement of the benzene ligand in the MD simulations. Figure S6 shows that the ligand rmsd fluctuates between 0.3 and 4.2 Å, reflecting that the symmetric ligand rotates freely in the binding site (in two of the 100 ns simulations, the ligand rmsd occasionally stabilizes around 3 Å). However, it never leaves the binding site. Therefore, it seems meaningless to restrict the averaging to certain structures; excluding some initial parts of the simulations as equilibration also has a minimal effect on the calculated entropies (Figure S7). Extrapolating the IE entropy^54^ according to
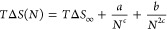
6gives reasonably consistent results for *c* = 0.2–0.3, –*T*Δ*S* = 15–16 kJ/mol (Table S2).

**Figure 9:**
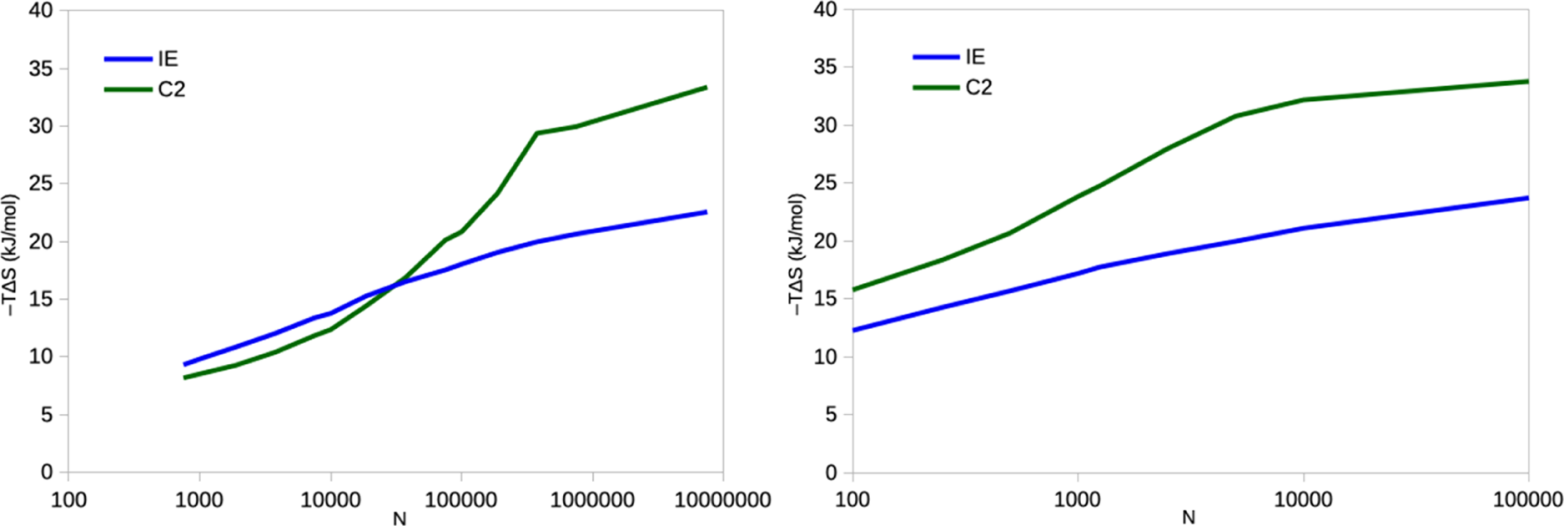
Entropies estimated by the IE and C2 methods for the binding of phenol to ferritin, estimated from 10 × 10 ns simulations with a sampling frequency of 10 fs (left) and 10 × 100 ns simulations with a sampling of 10 ps (right). In both cases, the simulations are then divided into batches of different sizes. Note the logarithmic scale on the *x*-axis.

The corresponding results for ferritin are shown in Figure 9. It can be seen that the IE entropy shows the same increasing trend with *N* as for lysozyme, but the variation is larger in absolute terms, 11–13 kJ/mol. The increase is essentially linear on the logarithmic scale. This variation is slightly larger than expected from Gaussian distributed data, 9–10 kJ/mol.

**Figure 10:**
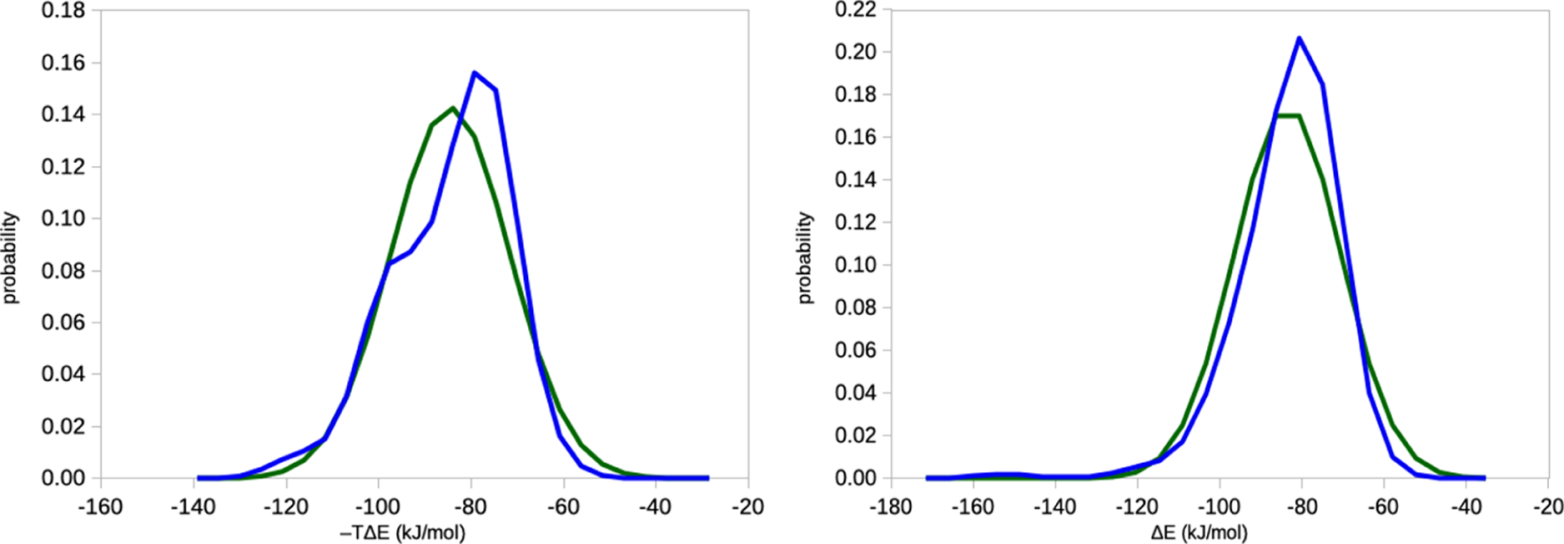
Distribution of the Δ*E*_IE_ energies for the binding of phenol to ferritin, estimated from 10 × 10 ns simulations with a sampling frequency of 10 fs (left) and 10 × 100 ns simulations with a sampling frequency of 10 ps (right). The green curve shows the ideal Gaussian distribution with the same average and standard deviation.

However, for ferritin, the C2 entropy also shows an increasing trend with *N*. In fact, the variation is larger than that for the IE entropy, 18–25 kJ/mol and with a more irregular trend. This reflects that σ_IE_ shows a similar increasing trend, 9–13 kJ/mol for the long simulation and 6–13 kJ/mol for the shorter simulation. This indicates that the data are distinctly non-Gaussian, as also seen in Figure 10 (with Gaussian data, there should be essentially no variation of the C2 entropy, <0.03 kJ/mol).

As for ferritin, the IE entropies estimated from the short simulations are lower than those from the long simulations, obtained from the same *N* (by 5–7 kJ/mol). Increasing the sampling frequency to 0.1 and 1 ps decreases the difference to 3 and 0.4 kJ/mol, respectively. This time, the statistical inefficiency is ∼40. Again, this indicates that a sampling frequency of 10 fs is too dense.

The rmsd of the ligand compared to the starting crystal structure is quite large and showing irregular fluctuations between 0.5 and 5 Å. Again, this reflects rotations of the small ligand in the binding site and no unbinding of the ligand. Such rotations are supported by the crystal structure, which shows two conformations of the ligand.^34^ Again, this makes it meaningless to restrict the averaging to certain structures. Removing the initial part of the simulation has a somewhat larger effect on ferritin than that on lysozyme (Figure S9), up to 5 and 7 kJ/mol for IE and C2 entropies, respectively, but decreasing with *N*. Extrapolation of the entropies with eq 6 also works worse than that for lysozyme, giving 26–29 kJ/mol for IE and 39–43±4–7 kJ/mol for the C2 entropies, based on the 100 ns simulations and *c* = 0.2–0.3 (Table S3).

## Conclusions

In this study, we have made a critical evaluation of the interaction-entropy method^23^ and the related approach involving a cumulant expansion, truncated at the second order,^22^ as cheap estimates of the entropies for MM/GBSA calculations. By employing simulations with Gaussian-distributed random numbers, we illustrate the extremely poorly conditioning of the exponential average, which is involved in the IE method. If the standard deviation of the Δ*E*_IE_ energies, σ_IE_, is larger than 15 kJ/mol, it becomes practically impossible to obtain converged results. Even worse, it is hard to recognize the problem because the extreme energies that determine the true value of the exponential average become very unlikely (cf. Figure 5). However, a good indication of the poor convergence (besides the large value of σ_IE_) is the steadily increasing value of the estimated entropy as the number of energies included in the exponential average increases, as shown in Figure 2. Several previous studies have pointed out the poor convergence of exponential averages for free-energy estimates and suggested various methods to decide whether the estimate is accurate,^54−58,60^ Theoretically, the C2 method shows much better convergence, up to σ_IE_ = 150 kJ/mol. However, for σ_IE_ > 25 kJ/mol, C2 entropies are too large to be realistic for a binding ligand.

Clearly, this is a practical problem because approximately half of the protein–ligand systems studied in the original IE and C2 publications^22,23^ give σ_IE_ > 15 kJ/mol. Moreover, 13% of the systems studied by Minh and co-workers and two of our studied systems give σ_IE_ > 25 kJ/mol. This was also the case for 13 protein–protein interactions.^24^ In that case, it was also observed that C2 entropies were much larger IE entropies. However, the authors suggested that “the Gaussian distribution obtained from relatively short MD runs...does not accurately represent the true energy distribution.” Therefore, they preferred the IE entropies, in contrast to our Gaussian simulations, which show that if IE and C2 entropies differ for large σ_IE_, the C2 results should be trusted more than those from IE. On the other hand, for alanine scanning calculations, the effect of the entropy seems to be relatively small.^25−27^ In one study, it was shown that predictions by IE and C2 typically agreed with a mean absolute deviation of 1 kJ/mol and maximum deviations of 9 kJ/mol.^25^

Zhang and co-workers suggested that the problem can be solved by using an extremely frequent sampling (every 10 fs). Our results indicate that this is too dense, giving strongly correlated energies. This does not affect the calculated entropies, but it is inefficient; for lysozyme and ferritin, the statistical inefficiency is 3–40, indicating that a sampling frequency of 0.1 ps would be more appropriate.

Moreover, both Zhang and Minh and co-workers suggested that the protein should be kept restrained during the simulations.^23,59^ This reduces σ_IE_ by 1–16 kJ/mol (7 kJ/mol on average) but of course also reduces the calculated entropy, which is not necessarily innocent, especially as the reduction varies between different proteins (so that the relative entropy also changes). The effect of the restraints on the dynamics and the other energy terms in eq 1 are also significant.^59^

**Figure 11:**
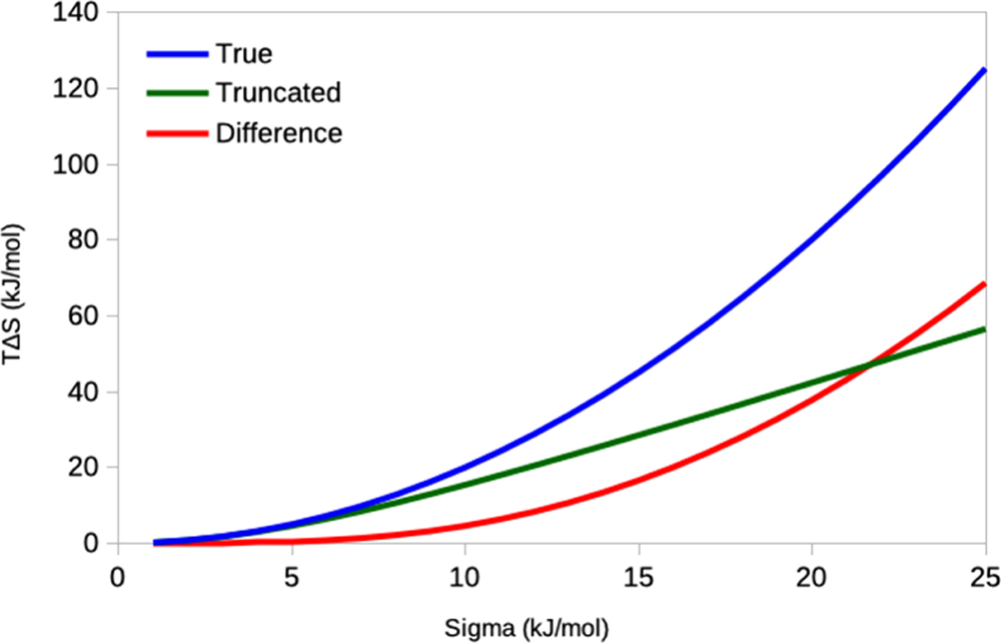
Effect of ignoring Δ*E*_IE_ values outside 3σ_IE_ when calculating *T*Δ*S*_IE_, according to Gaussian simulations. “True” is the analytical result, whereas “Truncated” is the results obtained from IE, using eq 3 and ignoring Δ*E*_IE_ values that deviate by more than 3σ_IE_ from the average. “Difference” is the difference between the two curves, that is, the error caused by the cutoff.

Moreover, Zhang and co-workers suggested that a cutoff should be employed for the IE energies, so that those >3σ_IE_ are ignored in the average of eq 3.^24^ From a statistical point of view, this is very questionable. As shown in Figure 5, when σ_IE_ is large, the correct Δ*G* or *T*Δ*S* is completely dominated by the lowest values of Δ*E*_IE_. If these are ignored or truncated, the calculated entropy will simply be incorrect (although the convergence to the wrong value will improve). Again, this can be illustrated by a Gaussian simulation, as shown in Figure 11. When σ_IE_ < 6 kJ/mol, Δ*E*_IE_ values outside 3σ_IE_ have little influence on the calculated entropy. However, for larger values, the truncated IE estimate rapidly diverges from the analytic results, giving a linear, rather than exponential increase with σ_IE_. The deviation is 5, 17, 38, and 69 kJ/mol at σ_IE_ = 10, 15, 20, and 25 kJ/mol, respectively.

Unfortunately, our applications on lysozyme and ferritin show that the convergence is worse than expected from the Gaussian model. For lysozyme (with σ_IE_ = 6 kJ/mol), C2 gives converged energies that do not depend on the sampling frequency. However, the IE energies are larger and increase steadily with *N*, although the variation is rather small (up to 6 kJ/mol). For ferritin (with σ_IE_ = 13 kJ/mol), both IE and C2 give entropies that increase steadily with *N* and with variations of up to 13 and 25 kJ/mol for *N* between 100 and 10,000,000. This may be caused by the fact that the Δ*E*_IE_ energies do not follow a Gaussian distribution. However, it can also reflect that as the simulations are elongated, more and more conformational states become available (higher activation barriers can be passed), as has been discussed before.^51^

There is a clear relation between σ_IE_ and the properties of the protein–ligand complexes: σ_IE_ is lower in systems where the ligand binds in a buried binding site, than when it binds on the surface. This natural and intuitive: in a solvent-exposed binding site, the ligand most likely retains much of its flexibility and group-rotation degrees of freedom, whereas inside the protein, they may be strongly restricted. However, σ_IE_ also seems to increase for charged ligands, simply because the interaction energies increase in magnitude, which is less obvious.

In conclusion, this study gives a rather pessimistic view of the applicability of IE or C2 entropies for MM/GBSA, except for alanine screening. Our results show that σ_IE_ should always be reported when using these methods. Moreover, it is advisable to calculate both IE and C2 entropies for the available data and to study how they depend on *N* by block averaging (which also provide an estimate of the precision of the calculated entropies). In addition, a sampling frequency of 10 fs seems to be 3–40 times too dense. Clearly, the IE method should be avoided if σ_IE_ > 15 kJ/mol because it is impossible to converge the exponential average. Moreover, C2 seems to give unrealistically large entropies when σ_IE_ > 25 kJ/mol. However, in practice, even when σ_IE_ < 15 kJ/mol, both methods often seem to have problems to give converged results. Still, it seems that relative entropies between similar ligands binding to the same protein are more stable, as shown in Figure 3, although the results are far from quantitative. Thus, estimating entropies from MD simulations remains a challenging task.

## Supporting Information Available

Absolute and relative entropies for the binding of three ligands to galectin-3 from 10 × 10 ns simulations with a sampling frequency of 10 fs; entropies estimated for the same system after 51 ns of equilibration were removed or after exclusion of snapshots with ligand RMSD > 2.2 Å, as well as ligand rmsd during the simulations and extrapolated values of the IE entropy; ligand rmsd, extrapolated IE entropies, as well as IE and C2 entropies estimated after 6 or 51 ns of equilibration were removed for the binding of benzene to lysozyme; ligand rmsd, extrapolated IE and C2 entropies, as well as IE and C2 entropies estimated after 4.25 or 51 ns of equilibration were removed for the binding of benzene to lysozyme. (PDF)
