# Supplementary material for: On the Use of Interaction Entropy and Related Methods to Estimate Binding Entropies
Source: J Chem Theory Comput. 2021 Jul 13;17(8):5379–91. doi: 10.1021/acs.jctc.1c00374 (PMC8389774; doi:10.1021/acs.jctc.1c00374)
Supplement: Supplementary file 1 — ct1c00374_si_001.pdf [file ct1c00374_si_001.pdf]

Supplementary Material

On the use of interaction entropy and related methods to estimate binding entropies

Vilhelm Ekberg and Ulf Ryde *

Department of Theoretical Chemistry, Lund University, Chemical Centre, P. O. Box 124, SE-221 00 Lund, Sweden

Correspondence to Ulf Ryde, E-mail: Ulf.Ryde@teokem.lu.se,

Tel: +46 – 46 2224502, Fax: +46 – 46 2228648

2021-07-04

Figure S1. Entropies estimated by the IE (left) and C2 (right) methods for the binding of three ligands to galectin-3 as a function of *N* from the 10 × 10 ns simulations with a sampling frequency of 10 fs. Note the logarithmic scale on the *x* axis.

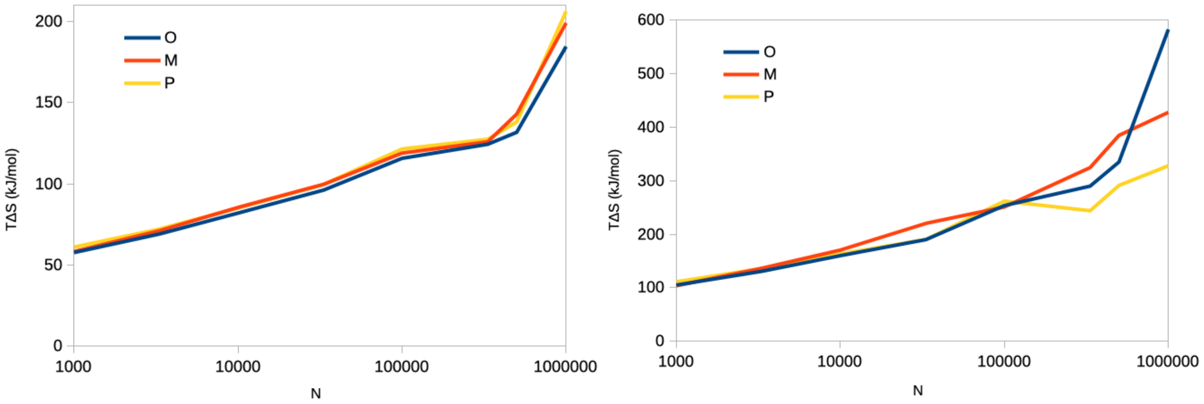

Figure S2. Relative entropies (defined as the entropy of each ligand minus the average of the three ligands) estimated by the IE (left) and C2 (right) methods for the binding of three ligands to galectin-3, from the 10 × 10 ns simulations with a sampling frequency of 10 fs.

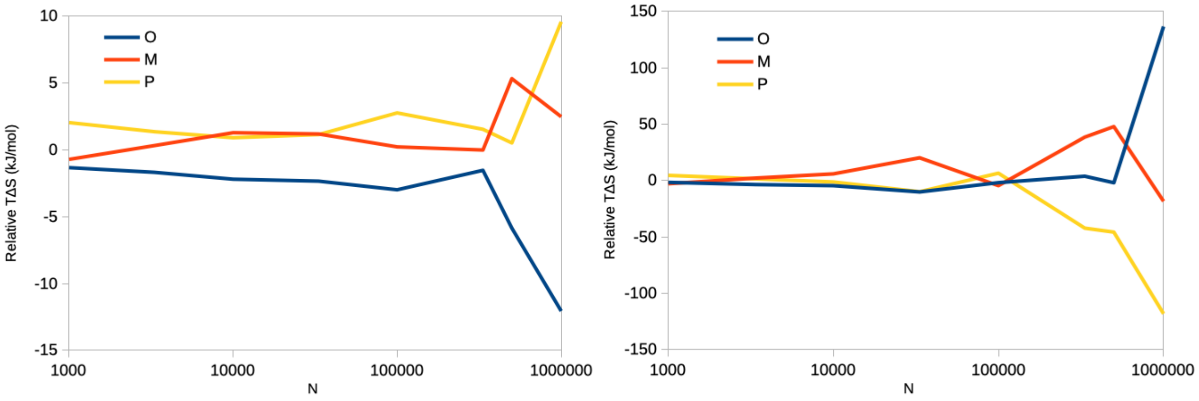

Figure S3. Entropies estimated by the IE (left) and C2 (right) methods by block averaging for the binding of three ligands to galectin-3 as a function of *N* from the 10 × 100 ns simulations after 51 ns of equilibration were removed. Note the logarithmic scale on the *x* axis.

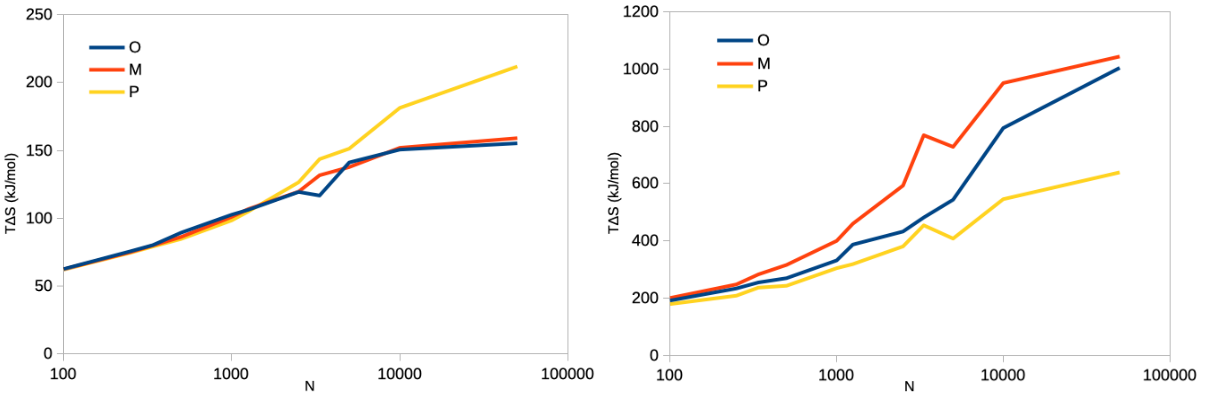

Figure S4. Root-mean-squared deviation (RMSD) of the ligand from the starting (crystallographic) conformation during the 10 x 100 and 10 x 10 ns MD simulations of the three ligands to galectin-3.

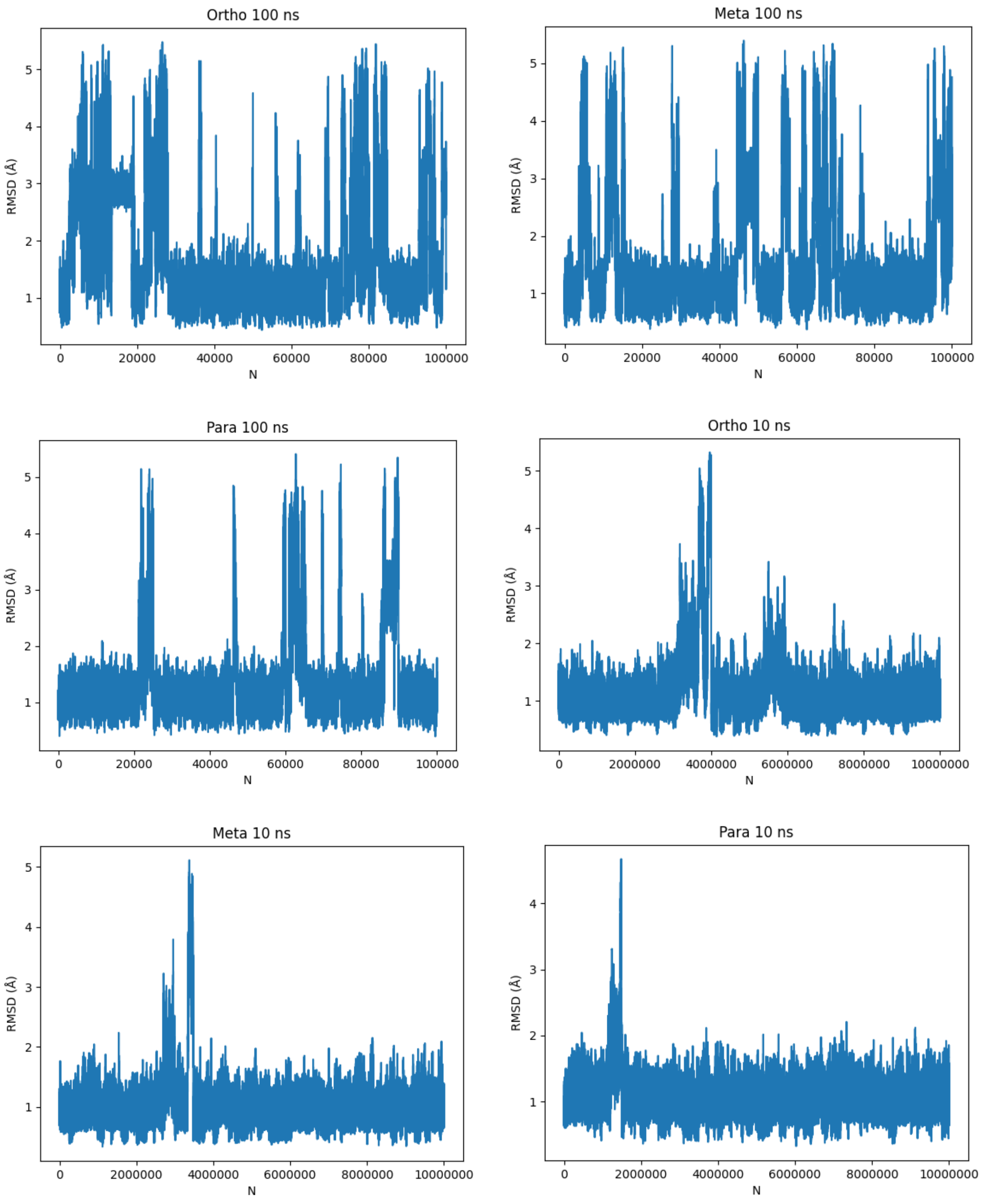

Figure S5. Entropies estimated by the IE (left) and C2 (right) methods by block averaging for the binding of three ligands to galectin-3 as a function of *N* from the 10 × 100 ns simulations after exclusion of snapshots with ligand RMSD > 2.2 Å. Note the logarithmic scale on the *x* axis.

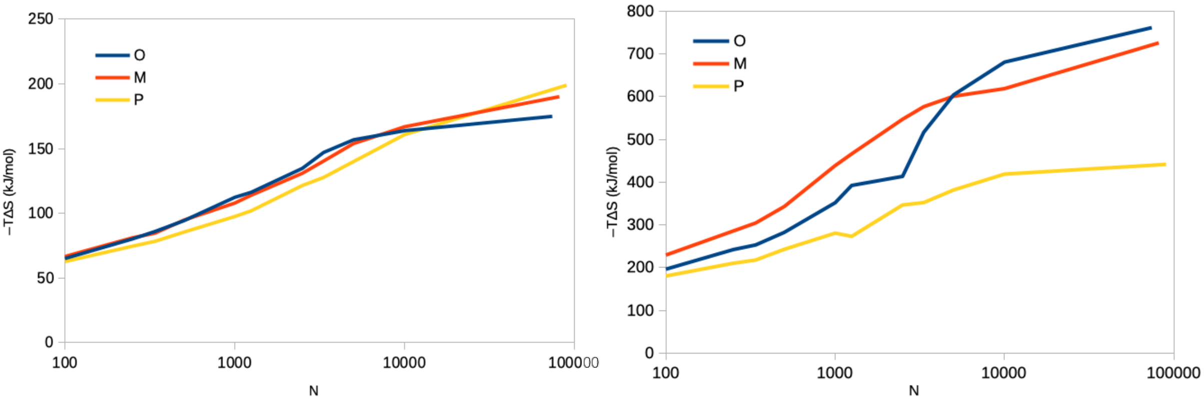

Table S1. Extrapolated values of the IE entropy for the binding of three ligands to galectin-3 as a function of *N* from the 10 × 100 ns simulations, employing Eq. 6.[54] with different values of *c*. $T\Delta S_{IE\infty}$ is reported with a 95% confidence interval in kJ/mol.

c	O	M	P
0.20	282±17	312±26	328±35
0.25	241±14	261±21	261±21
0.30	216±13	230±19	235±24
0.50	168±11	173±15	173±18

Figure S6. Root-mean-squared deviation (RMSD) of the ligand from the starting (crystallographic) conformation during the 10 x 100 and 10 x 10 ns MD simulations of the benzene in lysozyme.

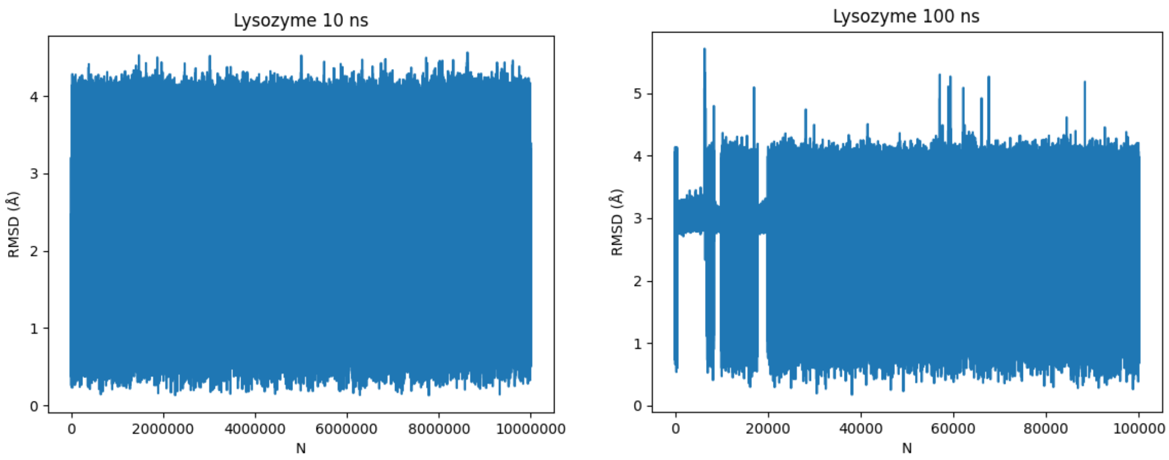

Figure S7. Entropies estimated by the IE and C2 methods by block averaging for the binding of benzene to lysozyme as a function of N from the 10 × 10 ns (left) or 10 × 100 ns (right) simulations after 6 or 51 ns of equilibration were removed. Note the logarithmic scale on the x axis. The calculated entropies agree with those obtained with only 1 ns equilibration within 0.5 and 0.1 kJ/mol for IE and C2, respectively.

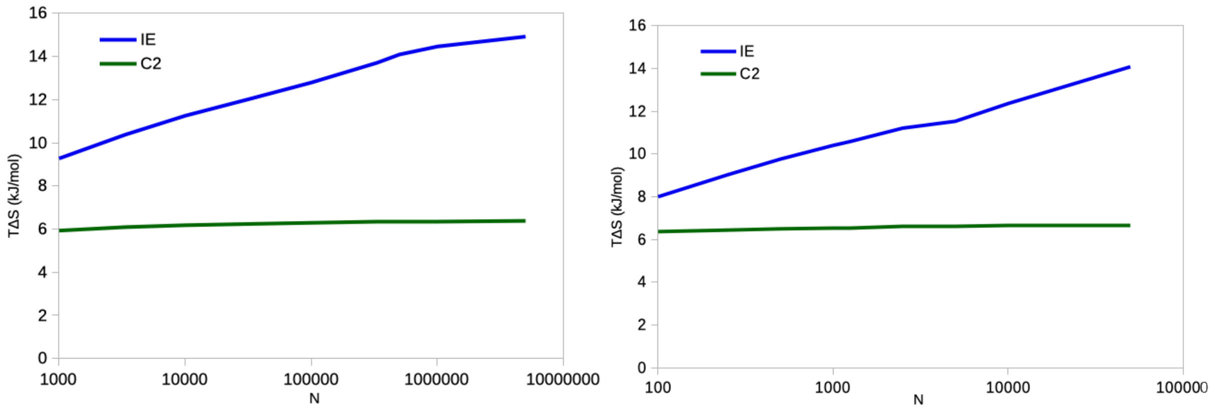

Table S2. Extrapolated values of the IE entropy for the binding of benzene to lysozyme as a function of N from the 10 × 100 ns simulations, employing Eq. 6.[54] with different values of c. $T\Delta S_{IE\infty}$ is reported with a 95% confidence interval in kJ/mol.

c	$T\Delta S_{IE\infty}$
0.20	16.4±0.6
0.25	15.6±0.5
0.30	14.7±0.4
0.50	13.1±0.6

Figure S8. Root-mean-squared deviation (RMSD) of the ligand from the starting (crystallographic) conformation during the 10 x 100 and 10 x 10 ns MD simulations of phenol in ferritin.

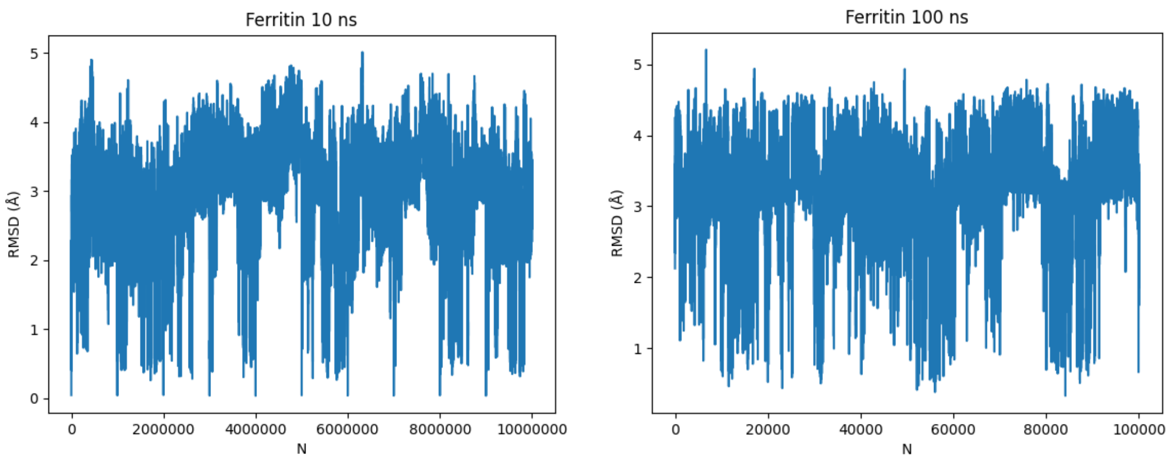

Figure S9. Entropies estimated by the IE and C2 methods by block averaging for the binding of benzene to lysozyme as a function of *N* from the 10 × 7.5 ns (left) or 10 × 100 ns (right) simulations after 4.25 or 51 ns of equilibration were removed. Note the logarithmic scale on the *x* axis.

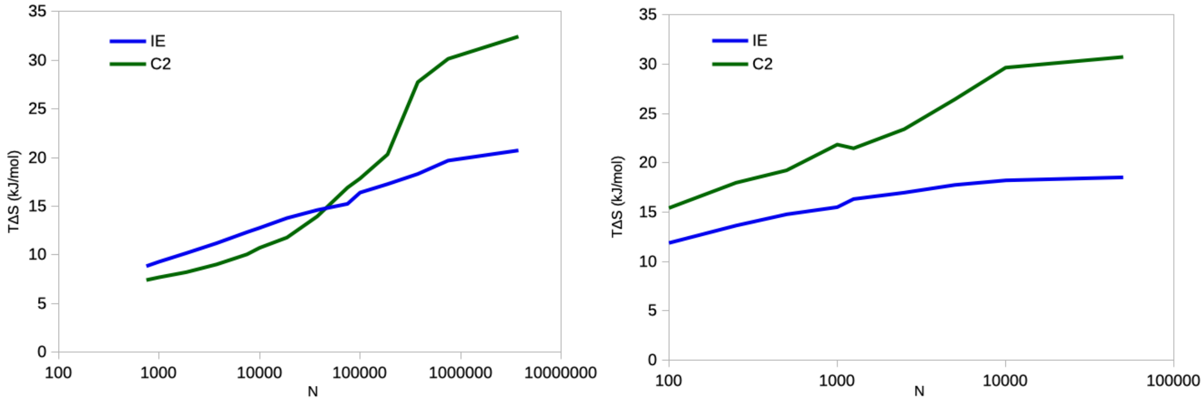

Table S3. Extrapolated values of the IE entropy for the binding of phenol to ferritin as a function of *N* from the 10 × 100 ns simulations, employing Eq. 6.[54] with different values of *c*. $T\Delta S_{\infty}$ is reported with a 95% confidence interval in kJ/mol.

c	$T\Delta S_{\infty}$	
	IE	C2
0.20	28.9±0.6	43±7
0.25	27.7±0.6	42±5
0.30	26.0±0.7	39±4
0.50	23.1±0.9	35±2
